# Conference Calendar

**DOI:** 10.1002/cfg.413

**Published:** 2004-07


**Comparative and Functional Genomics**
*Comp Funct Genom* 2004; **5**: 445–447.
Published online in Wiley InterScience (www.interscience.wiley.com). **DOI:** 10.1002/cfg.413

### Conference Calendar

November 2004–January 2005

### Animal models

Chicken Genome Sequence: Impact and Applications Symposium, 13–14 November

**http://www.avigenics.com/symposium1.htm**

Plant and Animal Genome XIII Conference, 15–19 January

**http://www.intl-pag.org**

### Bacteria

IP — Pathogens and Their Ecosystems, 17–20 November

**http://www.pasteur.fr/infosci/conf/sb/CLP5/**

ESF-FG — Environmental Genomics and Environmental Metagenomics, 18–20 November

**http://www.eez.csic.es/esf/**

1st International Conference on Environmental, Industrial and Applied Microbiology (BioMicro World2004), 13–16 December

**http://www.formatex.org/biomicroworld2004/index.htm**

### Bioinformatics

CODATA — 19th International Conference: The Information Society: New Horizons for Science, 7–10 November

**http://www.codata.org/04conf/index.html**

CSHL — Genomics Workshop: Identification of Functional Elements in Mammalian Genomes, 11–13 November

**http://meetings.cshl.org/2004/2004meetings.htm**

WSEAS — 5th International Conference on Mathematics and Computers in Biology and Chemistry, 15–17 November

**http://www.worldses.org/conferences/2004/iran/mcbc/index.html**

International Conference on Genome Informatics (GIW 2004), 13–15 December

**http://giw.ims.u-tokyo.ac.jp/giw2004/**

International Conference on Bioinformatics and Its Applications (ICBA'04), 16–19 December

**http://polaris.nova.edu/MST/ICBA2004/**

Pacific Symposium on Biocomputing, 4–8 January

**http://psb.stanford.edu/**

Asia–Pacific Bioinformatics Conference, 17–20 January

**http://www.deakin.edu.au/phoebe/APBC2005/**

### Evolution/comparative genomics

SICB — Annual Meeting, 4–8 January

**http://www.sicb.org/meetings/2005/index.php3**

### Functional genomics

CHI — Discovery on Target: Functional Genomics, 9–10 November

**http://www.healthtech.com/2004/fgn/index.asp**

IBC — EuroTIDES 2004, 22–24 November

**http://www.ibc-lifesci.com/eurotides/**

Copyright © 2004 John Wiley & Sons, Ltd.

### Human genomics and genetics

HUGO — 5th HUGO Pacific Meeting and 6th Asia–Pacific Conference in Human Genetics, 17–20 November

**http://www.hugopacific.com/**

### General

ASCB — 44th Annual Meeting, 4–8 December

**http://www.ascb.org/meetings/am2004/main04mtg.htm**

### Metabolomics

CHI — Metabolic Profiling, 13–15 December

**http://www.healthtech.com/conference-fall04.asp**

CHI — Drug Metabolism, 13–15 December

**http://www.healthtech.com/conference-fall04.asp**

### Pharmacogenomics

CHI — Discovery on Target: Target Validation, 11–12 November

**http://www.healthtech.com/2004/tvd/index.asp**

CHI — Safety/Toxicity Biomarkers, 15–16 November

**http://www.healthtech.com/2004/bmx/index.asp**

CHI — Fluorescent Proteins in Drug Development, 15–16 November

**http://www.healthtech.com/2004/gfp/index.asp**

CSHL/WT — Pharmacogenomics, 18–21 November

**http://meetings.cshl.org/2004/2004pharm.htm**

KEY — Meeting the Challenges of Drug Discovery, 15–20 January

**http://www.keystonesymposia.org/Meetings/ViewMeetings.cfm?MeetingID=719**

### Plants

CSHL — Plant Genomes: From Sequence to Phenome, 9–12 December

**http://meetings.cshl.org/2004/2004plants.htm**

Plant and Animal Genome XIII Conference, 15–19 January

**http://www.intl-pag.org**

### Proteomics

1st Latin American Protein Society Meeting, 8–12 November

**http://www.lnls.br/principal.asp?idioma=2&conteudo=450&opcaoesq=85**

CHI — PepTalk: The Protein Information Week, 10–13 January

**http://www.chi-peptalk.com/**

### Structural genomics

EMBO — Conference on Structures in Biology, 10–13 November

**http://www.embl-heidelberg.de/conferences/StructBiol04/**

3rd International Conference on Structural Genomics, 17–21 November

**http://www-nmr.cabm.rutgers.edu/icsg2004/**

KEY — Frontiers of NMR in Molecular Biology IX, 29 January–4 February

**http://www.keystonesymposia.org/Meetings/ViewMeetings.cfm?MeetingID=752**

Copyright © 2004 John Wiley & Sons, Ltd.

### Systems biology

BS — Systems Biology: Will It Work?, 12–14 January

**http://www.biochemistry.org/meetings/programme.cfm?Meeting_No=SA038**

### Transcriptomics

Critical Assessment of Microarray Data Analysis (CAMDA 2004), 10–12 November

**http://www.camda.duke.edu/camda04**

BS/RSC — RNA Structure and Function, 4–6 December

**http://www.biochemistry.org/meetings/programme.cfm?Meeting_No=SA031**

KEY — Diverse Roles of RNA in Gene Regulation, 8–14 January

**http://www.keystonesymposia.org/Meetings/ViewMeetings.cfm?MeetingID=749**

### Biotech industry

Bio-Europe 2004, 8–10 November

**http://www.ebdgroup.com/bioeurope/**

BioNorth 2004, 29 November–1 December

**http://www.bionorth.ca**

### Key

ASCB — American Society for Cell Biology: **http://www.ascb.org/**
BS — Biochemical Society: **http://www.biochemistry.org/**
CHI — Cambridge Healthtech Institute: **http://www.healthtech.com**
CODATA — The International Council for Science Committee on Data for Science and Technology: **http://www.codata.org/**
CSHL — Cold Spring Harbor Laboratories: **http://meetings.cshl.org/index.htm**
EMBO — European Molecular Biology Organization: **http://www.embo.org/**
ESF-FG — European Science Foundation Programme in Functional Genomics: **http://www.functionalgenomics.org.uk/**
HUGO — Human Genome Organization: **http://www.gene.ucl.ac.uk/hugo/**
IBC — IBC Conferences: **http://www.ibc-lifesci.com**
IP — Institut Pasteur: **http://www.pasteur.fr/**
KEY — Keystone Symposia: **http://www.keystonesymposia.org/**
RSC — Royal Society of Chemistry: **http://www.rsc.org/**
SICB — Society for Integrative and Comparative Biology: **http://www.sicb.org/**
WSEAS — World Scientific and Engineering Academy and Society: **http://www.worldses.org**
WT — Wellcome Trust: **http://www.wellcome.ac.uk/**

The *Conference Calendar* is a listing of forthcoming conferences of interest to our readership. We provide the title, dates and web address of each conference, to enable readers to find out more information. Inclusion does not imply endorsement by the journal. If you know of a relevant conference, please contact the Managing Editor.

Copyright © 2004 John Wiley & Sons, Ltd.

